# Sodium fluorescein in pediatric brain tumor surgery: a retrospective volumetric analysis of resection extent and consistency

**DOI:** 10.3389/fsurg.2026.1875864

**Published:** 2026-08-31

**Authors:** Robert Chrenko, Dana Kuniakova, Milan Liska, Martin Hanko, Alexandra Kolenova, Salome Jakesova, Pavol Kardos, Barbora Nedomova

**Affiliations:** 1 Department of Pediatric Neurosurgery, National Institute of Children’s Diseases, Bratislava, Slovakia; 2 Department of Neurosurgery, Faculty of Medicine, Slovak Medical University, Bratislava, Slovakia; 3 Medical Faculty, Comenius University, Bratislava, Slovakia; 4 Department of Neurosurgery,Slovak Medical University and University Hospital Bratislava, Bratislava, Slovakia; 5 Department of Radiology, National Institute of Children’s Diseases, Bratislava, Slovakia; 6 Department of Neurological and Spinal Surgery, Bory-Penta Hospitals, Bratislava, Slovakia; 7 Department of Pediatric Hematology and Oncology, Faculty of Medicine, National Institute of Children’s Diseases, Comenius University, Bratislava, Slovakia; 8 Department of Pediatric Anesthesiology and Intensive Care Medicine, Faculty of Medicine, National Institute of Children’s Diseases, Comenius University, Bratislava, Slovakia

**Keywords:** extent of resection (EOR), fluorescence-guided surgery, pediatric neuro-oncology, surgical consistency, volumetric magnetic resonance imaging

## Abstract

**Purpose:**

Maximizing the extent of resection (EOR) while preserving neurological function remains a key challenge in pediatric brain tumor surgery. Sodium fluorescein (SF) has been proposed as an adjunct to improve intraoperative visualization; however, evidence in pediatric populations is limited. This study evaluated the association between SF use and EOR, including the consistency of surgical outcomes.

**Methods:**

A retrospective single-center cohort of 58 pediatric patients (19 SF, 39 control) with intended gross total resection (GTR) was analyzed. EOR was assessed using postoperative volumetric MRI. Given the retrospective design and histological heterogeneity, analyses were considered exploratory.

**Results:**

The SF group showed greater mean EOR (99.67% ± 1.46 vs. 96.32% ± 11.53; *p* = 0.044), a higher GTR rate (94.7% vs. 61.5%; *p* = 0.011), and lower EOR dispersion (SD 1.46% vs. 11.53%; Levene's test *p* < 0.001). In exploratory histology-stratified analysis, these associations were mainly observed in pilocytic astrocytomas, whereas no relevant EOR difference was seen in non-pilocytic tumors.

**Conclusion:**

SF-guided surgery was associated with a modestly greater extent of resection and significantly higher GTR rate in selected pediatric brain tumors, predominantly pilocytic astrocytomas, with reduced EOR dispersion as a secondary finding. Given the retrospective design, limited sample size, and potential confounding—including the *post-hoc* nature of the GTR comparison—these findings are hypothesis-generating.

## Introduction

The application of intraoperative fluorophores in neurosurgery has been increasingly explored as an adjunct to enhance tumor visualization and support maximal safe resection (1). Among available agents, 5-aminolevulinic acid (5-ALA) and sodium fluorescein (SF) are the most commonly used in intracranial tumor surgery. While both have demonstrated utility in adult neuro-oncology, their role in pediatric populations remains less clearly defined, reflecting differences in tumor biology, blood–brain barrier characteristics, and surgical strategy (2–4).

In contrast to 5-aminolevulinic acid (5-ALA), whose usefulness in pediatric brain tumor surgery appears to be more limited and less predictable than in adult malignant glioma surgery, SF has been reported to provide a more consistent signal across a broader spectrum of lesions (5–7). This difference is likely multifactorial. As emphasized by Preuß et al., useful 5-ALA fluorescence in children appears to be observed mainly in high-grade gliomas, whereas medulloblastomas may fluoresce only inconsistently and most pilocytic astrocytomas remain non-fluorescent (2). This limited predictability probably reflects the distinct histological spectrum of pediatric brain tumors, in which low-grade gliomas and embryonal tumors are relatively frequent, as well as the different biological mechanisms of visualization: 5-ALA fluorescence depends on intracellular metabolic conversion to, and accumulation of, protoporphyrin IX in tumor cells. In contrast, SF accumulates predominantly through passive extravasation across a disrupted blood–brain barrier; accordingly, SF fluorescence intensity correlates with contrast enhancement on magnetic resonance imaging (MRI) (5, 8).

However, current evidence supporting the use of SF in children is largely derived from small, heterogeneous cohorts and feasibility-focused studies, limiting the generalizability of reported outcomes (9–13). Importantly, prior studies have predominantly focused on feasibility, safety, and surgeon-reported extent of resection (EOR), with limited use of objective volumetric MRI analysis (5, 7, 9, 12). Furthermore, the interpretation of EOR as a surrogate endpoint is inherently complex, as it reflects not only intraoperative visualization but also tumor characteristics, surgical intent, anatomical constraints, and adjunctive technologies (1). The marked heterogeneity of pediatric brain tumors—including differences between circumscribed and infiltrative lesions—further complicates the interpretation of aggregated results without tumor-specific stratification (14, 15).

Additionally, evidence from adult populations suggests that SF may be associated with improved rates of gross total resection (GTR) (16); however, these findings are largely derived from high-grade gliomas and metastases and cannot be directly extrapolated to pediatric cohorts. In children, where tumor biology and surgical decision-making differ substantially, the clinical relevance of modest differences in EOR remains uncertain.

In this context, we aimed to evaluate the association between SF use and surgical outcomes in pediatric brain tumors using objective volumetric MRI-based assessment. Rather than assuming a direct causal effect, we specifically examined whether SF use is associated with differences in (1) EOR and (2) dispersion of resection outcomes in cases with intended GTR.

Given the retrospective design, chronological allocation of patients, and histological heterogeneity, particular attention was given to potential sources of bias, including tumor-type distribution and temporal (era) effects. Accordingly, the findings of this study are intended to be hypothesis-generating rather than definitive.

## Methods

The study was approved by the Ethics Committee of the National Institute of Children's Diseases in Bratislava, Slovakia (approval date: April 29, 2025; approval number EK 08/4/2025). The requirement for informed consent was waived by the local Ethics Committee owing to the retrospective nature of the study. The study adhered to the Strengthening the Reporting of Observational Studies in Epidemiology (STROBE) guidelines (18).

### Study design and setting

This retrospective observational single-center study was conducted at the Department of Pediatric Neurosurgery in Bratislava, Slovakia, a tertiary care center serving a population of approximately 5.5 million. All pediatric patients meeting inclusion criteria and undergoing surgery between May 2019 and August 2024 were included. The SF group consisted of 19 pediatric patients (11 males and 8 females; mean age 8.58 ± 4.36 years), and the control group included 39 patients (21 males and 18 females; mean age 7.54 ± 4.60 years); baseline demographic characteristics are summarized in Table 1.

**Table 1:** Characteristics of the patients in the sodium fluorescein (SF) and control groups.

Variable	SF group (*n* = 19)	Control group (*n* = 39)	*p*-value
Study period	10/2022 to 08/2024	05/2019 to 09/2022	
Age (y) ± mean (SD)	8.58 ± 4.36	7.54 ± 4.60	0.408^a^
Sex (male:female)	11: 8	21:18	1.000^b^
Histology, *n* (%)			
Pilocytic astrocytoma	14 (73.74)	18 (46.15)	**0** **.** **056** ^b^
Medulloblastoma/PNET/embryonal tumor	3 (15.79)	13 (33.33)	0.217b
Ependymoma	2 (10.53)	1 (2.56)	0.248b
Other	0 (0.0)	7 (17.95)	0.083b
Location, *n* (%)			
Cerebellar	12 (63.15)	28 (71.79)	0.544^b^
Supratentorial superficial	4 (21.05)	8 (20.51)	1.000^b^
Supratentorial Intraventricular	3 (15.78)	2 (5.12)	0.318^b^
Intraoperative adjuncts, *n* (%)			
Microscope	19 (100)	39 (100)	1.000^b^
Neuronavigation	19 (100)	39 (100)	1.000^b^
CUSA	10 (52.63)	15 (38.46)	0.399^b^
IONM	4 (21.05)	20 (51.28)	**0** **.** **046** ^b^
USG	4 (21.05)	3 (7.69)	0.201^b^
5-ALA	0 (0.0)	2 (5.12)	1.000^b^

ALA, aminolevulinic acid; CUSA, Cavitron Ultrasonic Surgical Aspirator; IONM, intraoperative neurophysiological monitoring; PNET, primitive neuroectodermal tumor; SD, standard deviation; SF, sodium fluorescein; USG, ultrasound guidance.

a Welch's test.

b Fisher's exact test.

Bold text indicates statistical significance.

Group allocation was determined chronologically based on the availability of intraoperative fluorescence technology. From April 2019 to September 2022, patients underwent surgery under standard white-light microscopy (control group), whereas from October 2022 onward, following installation of the 560-nm filter, all eligible patients received SF (SF group). This non-randomized temporal allocation introduces a potential era effect, reflecting improvements in surgical experience, perioperative management, and institutional workflow over time.

### Inclusion criteria

Patients were included based on the following criteria:

1. age 0–18 years,
2. primary surgery,
3. histologically confirmed intracranial tumor,
4. documented preoperative surgical intent to achieve GTR, and
5. absence of concurrent intracranial pathology or neurological disease.

The preoperative intent to achieve GTR was determined from the documented surgical plan, based on multidisciplinary evaluation, including preoperative imaging, tumor location, radiological margins, presumed resectability, and absence of anticipated prohibitive factors such as extensive infiltration of eloquent structures or disseminated disease. Given the retrospective design, this determination relied on available clinical documentation rather than prospective standardization.

### Surgical procedures

Tumor resection was performed using standard microsurgical techniques with a Zeiss Kinevo 900 microscope (Zeiss, Oberkochen, Germany) and Medtronic neuronavigation system (Minneapolis, MN, USA). Additional intraoperative tools were used as indicated (Table 1). All resections were performed by one of four senior neurosurgeons, each with experience performing more than 100 brain tumor surgeries before April 2019.

### Patient grouping

Eligible patients were divided into two groups: 1) SF group, consisting of patients who underwent resection with 560-nm fluorescence guidance; and 2) control group, consisting of patients who underwent resection under standard white-light microscopy without SF guidance.

### Patient assignment

Patients were assigned to study groups based on the time period of surgery, reflecting the introduction of SF into routine clinical practice. Importantly, SF was administered to all eligible patients after October 2022, irrespective of tumor type, location, or radiological characteristics, thereby reducing selection bias at the level of fluorophore administration. However, differences in tumor-type distribution between groups may still reflect underlying referral patterns or temporal variability in case mix.

### Intraoperative fluorescence methodology

In the SF-guided group, a 560-nm filter was used with a Zeiss Kinevo 900 microscope to detect SF. SF (5 mg/kg) was administered intravenously by a trained anesthesiologist, with timing determined by the neurosurgeon at the start of tumor dissection.

The surgeon initially assessed tumor fluorescence under 560-nm light. Tumor resection in both groups was performed using standard white-light microscopy. In the SF-guided group, the surgeon intermittently applied 560-nm light to evaluate tumor fluorescence and distinguish the tumor tissue from normal brain. If deemed useful, fluorescence was intermittently applied until GTR was achieved and no tumor fluorescence remained.

Postoperatively, the surgeon recorded the utility of SF fluorescence as 1) useful, 2) partially useful, or 3) not useful. All participating surgeons were trained in intraoperative SF methodology before use.

### Outcome measures

The primary outcome was the EOR, calculated as:

EOR=(1−residualtumorvolume/initialtumorvolume)×100%

The EOR was determined using postoperative volumetric MRI analysis performed by an independent senior neuroradiologist with experience in pediatric neuro-oncology imaging. The assessor was not involved in the surgical procedures. Given the retrospective design and imaging characteristics, formal blinding to treatment group was not systematically feasible, although volumetric measurements were performed according to a standardized institutional protocol.

Tumor volume was assessed using manual slice-by-slice segmentation rather than linear three-plane measurements. Tumor boundaries were manually delineated on each relevant axial slice using the Siemens syngo.via software platform (Siemens Healthineers, Erlangen, Germany). Contrast-enhanced T1-weighted sequences were used when they best delineated tumor margins; in non-enhancing or poorly enhancing tumors, the MRI sequence providing the clearest tumor–brain interface was selected. Total tumor volume was calculated automatically by summation of the segmented cross-sectional areas multiplied by slice thickness. The same volumetric approach was applied to both preoperative and early postoperative MRI studies to calculate EOR.

Measurements were performed by a single experienced senior neuroradiologist with expertise in pediatric neuro-oncology imaging who was not involved in the surgical procedures. Given the retrospective design and imaging characteristics, formal blinding to treatment group was not systematically feasible, although measurements were performed according to a standardized institutional protocol.

Gross total resection (GTR) was defined as the absence of visible residual tumor on early postoperative MRI performed within 24–48 h after surgery. Residual tumor was assessed using the MRI sequence that best delineated tumor margins. In most contrast-enhancing lesions, this was contrast-enhanced T1-weighted imaging, interpreted together with standard T1-weighted, T2-weighted, FLAIR, DWI, and ADC sequences.

To enhance interpretability, EOR was also categorized as:

- gross total resection (GTR, 100%; no visible residual tumor on early postoperative MRI),
- subtotal resection (STR, 90%–99%), and
- partial resection (PR, <90%).

It should be noted that EOR reflects a composite endpoint influenced by tumor biology, anatomical constraints, and surgical strategy, and not solely intraoperative visualization.

### Secondary outcomes

Secondary outcomes included:

1. new postoperative neurological deficits at 6 months, assessed by independent neurologists,
2. functional outcome using the Lansky Play-Performance Scale (19), and
3. perioperative adverse events recorded descriptively, including potential SF-related complications.

Given the sample size, particularly in the SF group, the assessment of safety outcomes was considered exploratory and descriptive, without the statistical power to detect rare adverse events.

### Statistical analysis

Data were analyzed using IBM SPSS 26.0, JASP 0.19.1, and Microsoft Excel 2019. Continuous variables were compared using the Mann–Whitney *U* test due to non-normal distribution, and categorical variables were analyzed using Fisher's exact test. Effect sizes were calculated using rank-biserial correlation and phi coefficient, respectively. No adjustments for multiple confounders were performed due to sample size limitations.

Owing to the limited sample size and histological heterogeneity, tumor-specific subgroup analyses were not prespecified and were considered exploratory. Therefore, the results should be interpreted with caution, particularly regarding potential confounding by tumor type and surgical complexity.

To further explore the potential impact of histological imbalance, an exploratory subgroup analysis was performed stratifying tumors into pilocytic astrocytoma and non-pilocytic tumors. Within each subgroup, EOR was compared between SF-guided and control cases using the Mann–Whitney *U* test. Resection consistency was assessed descriptively using SD and interquartile range (IQR) of EOR.

## Results

### Volumetric analysis of the EOR and predictability of resection outcomes

At baseline, there was no significant difference in preoperative tumor volumes between the SF (38.12 ± 30.64 mL) and control (45.56 ± 31.14 mL; *p* = 0.394) groups, suggesting overall volumetric comparability. However, the SF group contained a higher proportion of pilocytic astrocytomas (73.74% vs. 46.15%, *p* = 0.056), whereas medulloblastomas/PNETs and ependymomas were more frequent in the control group, indicating a potential imbalance in tumor complexity between cohorts.

The SF group exhibited lower dispersion in EOR compared with the control group. A modest difference in mean EOR was also observed between groups (99.67% ± 1.46% vs. 96.32% ± 11.53%; *p* = 0.044, Mann–Whitney *U* test), although the clinical relevance of this difference remains uncertain.

**Figure 1:**
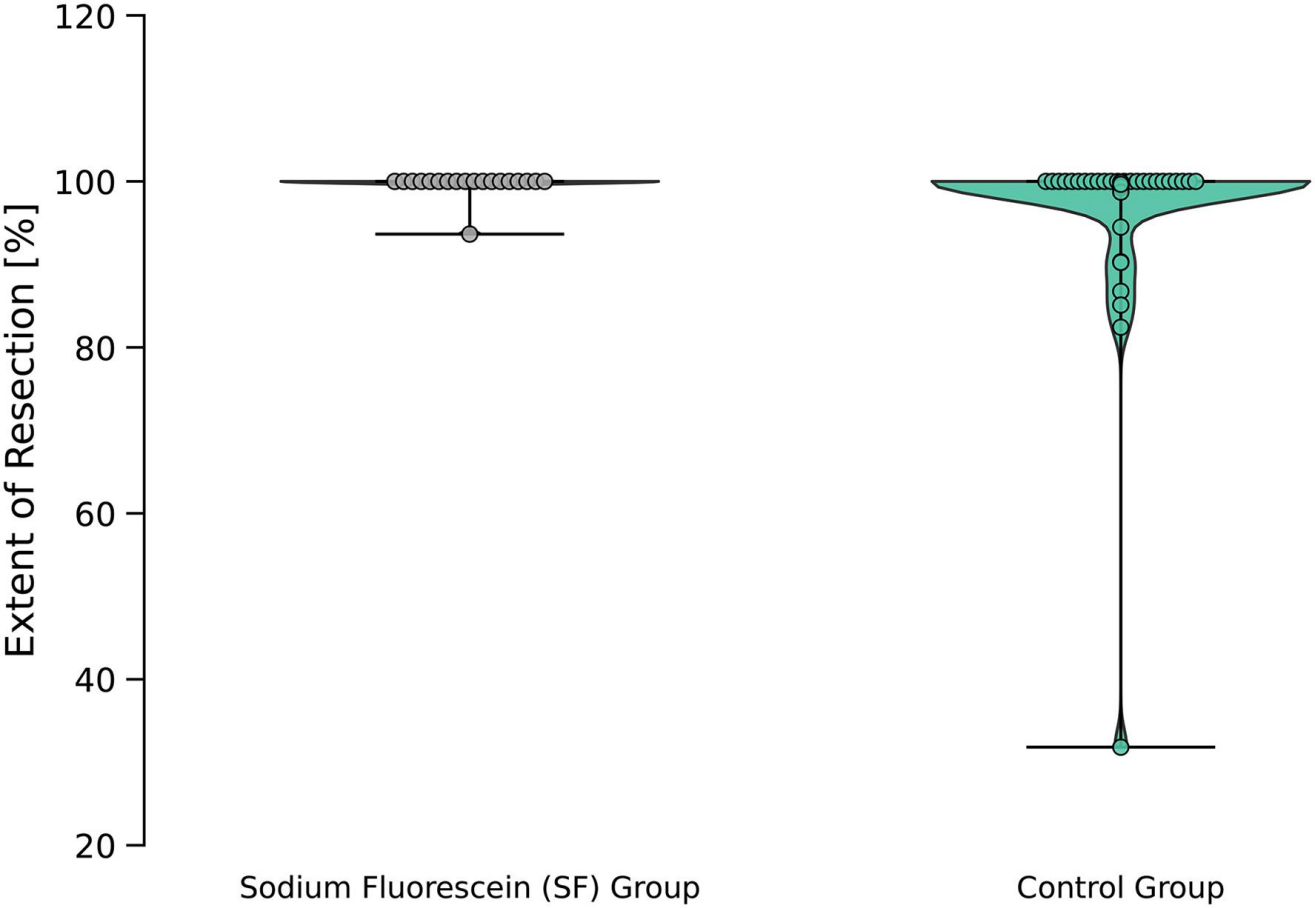
Extent of tumor resection in the SF and control groups. Comparison of the mean extent of tumor resection (EOR, %) assessed on postoperative magnetic resonance imaging between the patients in the sodium fluorescein (SF) and control groups. The *y*-axis shows the EOR (%). The mean EOR was higher in the SF group [99.67%; standard deviation (SD): 1.46] than in the control group (96.32%; SD: 11.53). Individual data points illustrate the distribution of the EOR values, demonstrating reduced dispersion in the SF group vs. the control group. One outlier with low EOR in the control group corresponded to a patient with a partially resected small (<1 mL) tumor with indistinct borders, without altering overall statistical significance.

The SF group also exhibited a significantly lower standard deviation of achieved EOR values compared with the control group (1.46% vs. 11.53%, *p* < 0.001, Levene's test), suggesting lower dispersion of resection outcomes (Figure 1). Postoperative residual tumor volumes were 0.27 ± 0.93 mL in the SF group and 0.59 ± 1.98 mL in the control group, without a statistically significant difference (*p* = 0.406).

To further explore the potential impact of histological imbalance, an exploratory subgroup analysis stratifying tumors into pilocytic astrocytoma (PCA) and non-pilocytic tumors was performed (Table 2). Within the PCA subgroup, the SF group demonstrated a higher mean EOR and markedly lower dispersion of achieved EOR values compared with controls (99.86% ± 0.36 vs. 93.54% ± 16.05; *p* = 0.041). In contrast, no relevant difference in EOR was observed between SF and control cases in the non-PCA subgroup (98.93% ± 2.76 vs. 98.74% ± 3.74; *p* = 0.901). Thus, the lower EOR dispersion observed in the overall cohort was mainly evident within the PCA subgroup and should be interpreted in the context of histological imbalance.

**Table 2:** Histology-stratified analysis of extent and consistency of resection of PCA and non-PCA tumors.

Variable	SF (*n* = 19)	Control (*n* = 39)	*p*-value
Pilocytic astrocytoma (PCA)			
*n*	14	18	
Extent of resection (%) ± mean (SD)	99.86 ± 0.36	93.54 ± 16.05	**0** **.** **041** ^c^
Median EOR (%) [IQR]	100 [100–100]	100 [94–100]	
Rate of GTR, *n* (%)	13 (92.86)	14 (77.78)	0.355^b^
Non-pilocytic tumors (non-PCA)			
n	5	21	
Extent of resection (%) ± mean (SD)	98.93 ± 2.76	98.74 ± 3.74	0.901^c^
Median EOR (%) [IQR]	100 [99–100]	100 [100–100]	
Rate of GTR, *n* (%)	5 (100.00)	10 (47.62)	0.053^b^

EOR, extent of resection; GTR, gross total resection; IQR, interquartile range; SD, standard deviation; SF, sodium fluorescein.

b Fisher's exact test.

c Mann–Whitney *U* test.

Bold text indicates statistical significance.

### Categorical resection outcomes

Categorical resection outcomes are shown as GTR, STR, and PR, with distributions of GTR:STR:PR = 18:1:0 in the SF group and 24:11:4 in the control group (Table 3).

**Table 3:** Tumor volume, extent of resection, and postoperative outcomes in the sodium fluorescein (SF) and control groups.

Variable	SF group (*n* = 19)	Control group (*n* = 39)	*p*-value
Volume of tumor preoperatively (mL) ± mean (SD)	38.12 ± 30.63	45.56 ± 31.14	0.394^a^
Volume of tumor postoperatively (mL) ± mean (SD)	0.27 ± 0.93	0.59 ± 1.98	0.406^a^
Extent of resection (%) ± mean (SD)	99.67 ± 1.46	96.32 ± 11.53	**0** **.** **044** ^c^
Extent of resection [*n*; GTR:STR:PR]	18:1:0	24:11:4	**0** **.** **029** ^b^
Rate of GTR, *n* (%)	18 (94.73)	24 (61.53)	**0** **.** **011** ^b^
Clinical outcome, mean (SD)			
PICU stay (d) ± mean (SD)	1.32 ± 0.48	2.69 ± 4.22	0.052^a^
Hospital stay (d) ± mean (SD)	6.79 ± 3.20	8.37 ± 4.28	0.123^a^
Follow-up (y) ± mean (SD)	0.70 ± 0.50	1.90 ± 1.06	**<0** **.** **001** ^a^
New neurological deficit developed at 6 mo, *n* (%)	3 (15.78)	11 (28.20)	0.347^b^
Lansky score at 6 mo (%), mean (SD)	96.84 ± 8.20	93.33 ± 10.59	0.298^c^

GTR, gross total resection; PICU, pediatric intensive care unit; N/A, not applicable; STR, subtotal resection; SF, sodium fluorescein; PR, partial resection; SD, standard deviation.

a Welch's test.

b Fisher's exact test.

c Mann–Whitney *U* test.

Bold text indicates statistical significance.

GTR was achieved in 94.73% (18/19) of patients in the SF group compared with 61.53% (24/39) in the control group (*p* = 0.011, Fisher's exact test), although this observation should be interpreted cautiously given the exploratory design and potential confounding factors. The STR and PR rates were 5.25% and 0% in the SF group vs. 28.20% and 10.25% in the control group, respectively.

Among patients with subtotal or partial resection at the initial operation, re-operation was performed in 3 of 15 patients in the control group (20.0%) and in 0 of 1 patient in the SF group (0%). All three re-operations in the control group were performed because of increasing residual tumor volume after discussion by the multidisciplinary tumor board. Given the very small number of subtotal resections in the SF group, this observation was considered descriptive only.

### Postoperative neurological deficits

New permanent neurological deficits at 6 months were observed in 3 of 19 patients in the SF group (15.78%) and in 11 of 39 patients in the control group (28.20%) (Table 3). To explore potential confounding factors, an exploratory descriptive stratified analysis of new neurological deficits at 6 months was performed. New deficits were less frequent in the SF group than in the control group, but this difference was not statistically significant (3/19, 15.8% vs. 11/39, 28.2%; Fisher's exact test *p* = 0.347).

In contrast, deficits were more frequent in non-PCA tumors than in PCA tumors (11/26, 42.3% vs. 3/32, 9.4%; *p* = 0.005) and in patients who received radiotherapy and chemotherapy than in those who did not (11/24, 45.8% vs. 3/34, 8.8%; *p* = 0.002). IONM use was not significantly associated with new neurological deficits (5/24, 20.8% vs. 9/34, 26.5%; *p* = 0.759). Because only 14 neurological events occurred, these analyses were considered descriptive and no multivariable regression model was performed.

### Functional outcomes

Functional outcomes assessed using the Lansky Play-Performance Scale at 6 months were similar between the groups. The mean score was 96.84% ± 8.20% in the SF group and 93.33% ± 10.59% in the control group (*p* = 0.298, Mann–Whitney *U* test) (Table 3).

### Usefulness of SF fluorescence

Intraoperative SF fluorescence was judged as useful or partially useful in 15 of 19 SF-guided cases (78.9%) and not useful in 4 cases (21.1%). Cases without useful fluorescence included one pilocytic astrocytoma, one ependymoma, and two medulloblastomas. In an exploratory comparison, SF usefulness appeared more frequent in pilocytic astrocytomas than in non-PCA tumors (13/14, 92.9% vs. 2/5, 40.0%; Fisher's exact test *p* = 0.037), although this finding should be interpreted cautiously because of the small subgroup size.

### Adverse effects of SF

Adverse effects of SF were monitored intraoperatively and within 24 h postoperatively. No major SF-related adverse events, including hypersensitivity or cardiopulmonary complications, were observed in this cohort.

Minor symptoms such as headache or gastrointestinal discomfort could not be reliably distinguished from the typical postoperative course. Transient yellow discoloration of the skin, sclera, and urine was observed but not systematically analyzed.

## Discussion

Advances in pediatric brain tumor management have introduced multiple intraoperative adjuncts aimed at maximizing safe resection while preserving neurological function (1, 17). Despite these developments, the EOR remains a multifactorial endpoint influenced by tumor biology, anatomical constraints, and surgical strategy rather than a single intraoperative variable.

In this exploratory retrospective cohort, the principal observation was a modestly greater extent of resection and a higher rate of GTR in the SF group, with reduced dispersion of EOR values as a secondary finding suggesting more consistent resection patterns. The direct clinical **relevance** of these associations—particularly with respect to long-term outcomes such as recurrence or survival—cannot be determined from the present dataset. These findings suggest that the association of SF guidance may relate both to the extent of resection achieved and to the consistency and predictability of surgical outcomes.

The lower dispersion of achieved EOR values in the SF group should not be interpreted as a validated clinical endpoint, but rather as an exploratory descriptor of resection consistency. In this cohort, all included cases had a documented preoperative intent to achieve GTR; therefore, lower dispersion may indicate more predictable achievement of the intended surgical goal and fewer unexpectedly incomplete resections. In cases where GTR is intended, an unplanned subtotal resection typically prompts multidisciplinary re-evaluation and may influence decisions regarding re-operation or adjuvant therapy; greater predictability of achieving the intended surgical goal may therefore carry downstream clinical relevance, although this was not directly assessed in the present dataset. However, the clinical relevance of this observation remains uncertain and requires confirmation in larger prospective cohorts.

The histology-stratified analysis showed that the lower dispersion of achieved EOR values was mainly observed in the PCA subgroup, whereas no comparable signal was evident in non-PCA tumors. This supports the reviewer's concern that tumor biology and case mix may have contributed to the overall finding. Pilocytic astrocytomas are typically more circumscribed and more amenable to complete resection than many embryonal or infiltrative tumors; therefore, the observed association between SF use and resection consistency should not be interpreted as a uniform effect across all pediatric brain tumor types.

These findings should be interpreted in the context of several important limitations. The cohort was histologically heterogeneous, and tumor-specific analyses were not feasible due to sample size constraints. Pediatric brain tumors differ substantially in their growth patterns, invasiveness, and surgical resectability, and the observed association between SF use and EOR may therefore be influenced by underlying tumor biology rather than fluorescence guidance alone. In particular, the higher proportion of pilocytic astrocytomas in the SF group, which are typically well circumscribed and more amenable to complete resection—represents a potential source of confounding.

The non-randomized, time-bound group allocation introduces a potential era effect, whereby improvements in surgical experience, perioperative care, and institutional workflow over time may have contributed to improved outcomes independently of SF use. Although all participating surgeons had substantial experience prior to the study period, incremental improvements in multidisciplinary care and operative strategy cannot be excluded.

All included cases were selected based on a preoperative intent to achieve GTR. While this approach allowed for a more standardized evaluation of EOR as a quantitative endpoint, it inherently excludes tumors in which subtotal resection is the appropriate surgical strategy (e.g., infiltrative brainstem lesions or disseminated disease). As a result, the findings are not generalizable to the full spectrum of pediatric brain tumors and may preferentially reflect cases with more favorable resectability profiles.

The sample size—particularly in the SF group—limits statistical robustness and precludes definitive conclusions regarding safety. Although no major SF-related adverse events were observed, this study is underpowered to detect rare complications, and safety conclusions should therefore be considered descriptive rather than definitive. The overall follow-up duration differed between groups (0.70 ± 0.50 years in the SF group vs. 1.90 ± 1.06 years in the control group; *p* < 0.001), this asymmetry does not affect the validity of the secondary neurological and functional outcomes reported in this study. All patients in both groups completed the prespecified 6-month postoperative assessment, at which point neurological status and Lansky Play-Performance Scale scores were recorded. The reported outcomes therefore reflect a uniform observation window across both cohorts, irrespective of subsequent clinical course or duration of longer-term follow-up in individual patients.

The comparison of re-operation after subtotal or partial resection was also limited by the markedly unequal number of incomplete resections between groups and by the shorter follow-up in the SF cohort. Therefore, the lower observed number of re-operations in the SF group was interpreted descriptively only and should not be considered evidence of an oncological benefit of SF.

Another methodological limitation concerns volumetric MRI assessment. Although manual slice-by-slice segmentation provides a more objective estimate of tumor volume than linear three-plane measurements, it remains susceptible to measurement bias, particularly in tumors with indistinct margins, postoperative changes, or heterogeneous enhancement. In addition, all measurements were performed by a single neuroradiologist, and interobserver reproducibility was not assessed.

The relationship between intraoperative fluorescence and tumor biology was not specifically addressed in this study. The mechanism of SF accumulation is based on blood–brain barrier disruption. The intraoperative usefulness of SF was not uniform across tumor types. In 4 of 19 SF-guided cases, fluorescence was judged as not useful. This most likely reflects tumor-related heterogeneity in blood–brain barrier disruption, contrast enhancement, and SF accumulation. Because SF was administered to all eligible patients after introduction of the 560-nm filter, irrespective of tumor type, location, or radiological characteristics, we do not consider these cases to represent selection bias at the level of fluorophore administration. However, non-uniform fluorescence usefulness across histological entities represents an additional limitation and should be addressed in larger tumor-specific studies.

A statistically significant imbalance in the use of intraoperative neurophysiological monitoring (IONM) was observed between groups (21.05% in the SF group vs. 51.28% in the control group; *p* = 0.046), representing an additional potential confounder. The higher use of IONM in the control group likely reflects perceived surgical complexity and case-mix characteristics during the earlier study period rather than a direct protective factor for the extent of resection. With increasing familiarity of the neurophysiology team and evolving case selection, IONM utilization stabilized over time, as reflected in the lower rates observed in the SF group.

Nevertheless, this imbalance cannot be fully excluded as a contributing factor to the observed differences in outcomes between cohorts.

In the stratified analysis, neurological deterioration was more frequent in non-PCA tumors and in patients requiring radiotherapy and chemotherapy, suggesting that postoperative neurological outcome was more closely related to tumor biology and overall treatment complexity than to SF guidance itself. Because only 14 neurological events occurred, multivariable regression was not considered statistically reliable and these findings were interpreted descriptively.

The present findings are consistent with previous reports describing the feasibility of SF-guided surgery in pediatric neurosurgery (5, 7, 9, 12, 13). However, unlike most prior studies, which relied on subjective or surgeon-reported outcomes, this exploratory study employed objective volumetric MRI analysis, providing a more rigorous assessment of resection extent and consistency. Nevertheless, the retrospective design and the presence of multiple confounding factors limit causal interpretation.

Taken together, the results of this exploratory volumetric observational study suggest that SF use was associated with a modestly greater and more predictable extent of resection. Its potential value may lie both in supporting a higher rate of complete resection and in reducing dispersion of surgical outcomes, particularly in cases where the distinction between tumor and normal brain tissue is less clear.

Future prospective, multicenter studies with larger cohorts, standardized imaging protocols, and tumor-specific stratification are needed to clarify the precise role of SF in pediatric neuro-oncology. In particular, studies incorporating long-term oncological outcomes and functional measures will be essential to determine whether improvements in resection extent and consistency translate into meaningful clinical benefit.

The findings of this study should therefore be interpreted within the context of methodological limitations and viewed as hypothesis-generating rather than definitive evidence.

## Conclusion

In this exploratory volumetric observational study, SF-guided surgery was associated with a modestly greater extent of resection and a higher rate of GTR, an effect most pronounced in pilocytic astrocytomas; SF use was also associated with reduced dispersion of EOR, a secondary finding suggesting more consistent attainment of the surgical goal. As the categorical GTR comparison was not prespecified, it should be regarded as descriptive rather than confirmatory. Because of the limited sample size, tumor heterogeneity, and retrospective design, these results should be interpreted cautiously and confirmed in prospective multicenter studies.

## Data Availability

The raw data supporting the conclusions of this article will be made available by the authors, without undue reservation.
